# Urologic surgery laparoscopic access: vascular complications

**DOI:** 10.1590/S1677-5538.IBJU.2015.0369

**Published:** 2017

**Authors:** Anibal Wood Branco

**Affiliations:** 1 Departamento de Urologia Hospital Cruz Vermelha, Curitiba, Brasil

## Abstract

Vascular injury in accidental punctures may occur in large abdominal vessels, it is known that 76% of injuries occur during the development of pneumoperitoneum. The aim of this video is to demonstrate two cases of vascular injury occurring during access in laparoscopic urologic surgery. The first case presents a 60-year old female patient with a 3cm tumor in the superior pole of the right kidney who underwent a laparoscopic partial nephrectomy. After the Verres needle insertion, output of blood was verified. During the evaluation of the cavity, a significant hematoma in the inferior vena cava was noticed. After the dissection, a lesion in the inferior vena cava was identified and controlled with a prolene suture, the estimated bloos loss was 300ml. The second case presents a 42-year old female live donor patient who had her right kidney selected to laparoscopic live donor nephrectomy. After the insertion of the first trocar, during the introduction of the 10mm scope, an active bleeding from the mesentery was noticed. The right colon was dissected and an inferior vena cava perforation was identified; a prolene suture was used to control the bleeding, the estimated blood loss was 200mL, in both cases the patients had no previous abdominal surgery. Urologists must be aware of this uncommon, serious, and potentially lethal complication. Once recognized and in the hands of experienced surgeons, some lesions may be repaired laparoscopically. Whenever in doubt, the best alternative is the immediate conversion to open surgery to minimize morbidity and mortality.

## ARTICLE INFO

Available at: http://www.intbrazjurol.com.br/video-section/branco_166_166

Int Braz J Urol. 2017; 43 (Video #1): 166-166

**Correspondence address:** Anibal Wood Branco, MD. Departamento de Urologia. Hospital Cruz Vermelha, Curitiba, Brasil. Avenida: Vicente Machado 1280. Curitiba, 80420011, Brasil. E-mail: anibal@awbranco.com.br

